# Population-Level Interest in Glucagon-Like Peptide-1 Receptor Agonists for Weight Loss Using Google Trends Statistics in a 12-Month Retrospective Analysis: An Infodemiology and Infoveillance Study

**DOI:** 10.7759/cureus.71569

**Published:** 2024-10-15

**Authors:** Laith G Shareef, Shahad Sabah Khalid, Mohanad Faris Raheem, Ali Fawzi Al-Hussainy, Noor Sameer Al-Khayyat, Ahmed Zakaria Al Arajy, Mustafa M Noori, Mohammad Alameen Qasim, Hanan Hussein Jasim

**Affiliations:** 1 Pharmacy Department, Al-Rasheed University College, Baghdad, IRQ; 2 Pharmacy Deparment, Al-Nukhba University, Baghdad, IRQ; 3 Clinical Biochemistry Department, Al-Bayan University, College of Pharmacy, Baghdad, IRQ; 4 Clinical Pharmacy Department, Ahl Al Bayt University, College of Pharmacy, Kerbala, IRQ; 5 Clinical Pharmacy Department, University of Mashreq, Faculty of Pharmacy, Baghdad, IRQ; 6 Pharmacy Department, Al-Farabi University College, Baghdad, IRQ; 7 Pharmaceutics Department, Mustansiriyah University, College of Pharmacy, Baghdad, IRQ; 8 Clinical Pharmacy Department, Al-Emamin Al-Kadhimeen Medical City Hospital, Baghdad, IRQ

**Keywords:** glp-1 agonists, google trends healthcare, infodemiology, mounjaro, obesity, ozempic, saxenda, social media, wegovy, weight loss

## Abstract

Obesity is an exacerbated public health challenge, increasing the risk of several diseases and mortality while deteriorating the quality of life. There is significant dedication to exploring obesity therapies using glucagon-like peptide-1 (GLP-1) agonists, which have shown efficacy in reducing the number of deaths and complications associated with type 2 diabetes. This research aimed to examine the recent search popularity of GLP-1 agonists using Google Trends at both national (in Iraq) and global levels. To quantify relative search volume (RSV), the total search query activity has been transformed to a percentage scale ranging from 0% to 100%. The word "Ozempic" was chosen because of its extensive coverage in social media and web/print publications pertaining to this subject matter. A comparative search was performed targeting the phrases "Wegovy," "Saxenda," and "Mounjaro" to identify a novel combination GLP-1 agonist from August 2023 to August 2024. The present study demonstrated a statistically significant difference in the RSV among the four drugs (P < 0.0001) nationally and globally. In Iraq, the highest RSV for Ozempic was documented in Duhok, followed by Sulaymaniyah and Erbil. A comparable RSV profile has been noted for Saxenda, while substantial interest in Wegovy is seen in Ninawa. Meanwhile, globally, the highest RSV for Ozempic was recorded in Canada, the United States of America, and Australia. A distinct RSV profile has been observed for Saxenda, with heightened search interest recorded in Latin America, Poland, Sweden, and Australia. By contrast, Mounjaro received search interest primarily in Greenland and the United States, while Mounjaro search interest was noted in Canada, the United States, and Australia. This study demonstrates a significant and growing public interest in GLP-1 agonists, namely, Ozempic, Saxenda, Wegovy, and Mounjaro. As the use of GLP-1 agonists for weight loss becomes more common, more knowledge, understanding, and continuous scientific research will make it more convenient to obtain the best patient outcomes.

## Introduction

Approximately 650 million individuals worldwide are affected by obesity, a chronic condition characterized by excessive adiposity that deteriorates health; various metabolic issues, including type 2 diabetes, metabolic-dysfunction associated steatotic liver disease, heart disease, arthritis, and obstructive sleeping apnea, are more likely to develop as a result of obesity [1-3]. Lifestyle modifications, such as eating habits, physical activity, and alterations in behavior, are crucial for managing obesity and provide many advantages; however, even the most rigorous lifestyle interventions only lead to an average reduction in weight of up to 10%, maintaining this weight remains difficult, as 80% of the reduction in weight is anticipated to be regained within the following five years [4].

Innovative hormone-based medications with exceptional efficacy for obesity are emerging; this is partly motivated by the recognition of obesity as a disease characterized by energy dysregulation and intricate underlying pathology, in which the energy homeostasis system sustains an increased fat mass [5]. Investigation of the function of hormones in regulating metabolism and appetitive behavior, as well as their physiological impact on cerebral energy control, has redirected the focus of obesity treatment toward targeting fundamental processes via the invention of hormone-based drugs. Glucagon-like peptide-1 (GLP-1) makes up one of the nutrient-stimulated pancreatic and intestinal hormones that provide appetitive signals, initially designed as medications for managing type 2 diabetes (T2D) via the action of its incretin. GLP-1 receptor agonists (GLP-1 RAs) are now becoming recognized as very successful treatments for obesity [6]. Studies have shown that GLP-1 agonists result in placebo-adjusted weight reduction ranging from 12% to 18%, surpassing any previous pharmacological treatment and attracting significant interest and application [7]. However, there is still uncertainty among clinicians and the general public over the actual expenses, acceptability, and availability. Fifty percent of people in the United States express interest in using a prescription weight-loss medication, and among this group, 93 million individuals satisfy the eligibility requirements for GLP-1 [7]. Despite standard methods continuing to be valuable for tracking trends, the accessibility of Internet data provides researchers with a more efficient method to monitor emerging trends, an area now known as infodemiology [8]. While social networking tracking shows potential for infodemiology, it can be challenging by social media platforms restricting or altering data access, the constant flux of the social media landscape, with fluctuating user numbers and novel apps (e.g., TikTok) emerging and attracting substantial user bases, and the evolving nature of content on social networks, shifting from mainly text to graphic content and finally video-posing new computing challenges; search engine data, such as Google Trends (GT), is an alternative and potentially valuable data source for infodemiology [9]. While the data from Internet searches may not be as extensive as that from social media, they still provide interesting insights into the motives of search users, when handled adequately [10]. The purpose of this study is to offer an in-depth analysis of national and global interest data associated with GLP-1 receptor agonists over a one-year period.

## Materials and methods

Study design

The current study presents an analysis of individuals' online search activity using the Google search engine for queries related to GLP-1 receptor agonists for weight loss during a period of one year, concentrated on the time frame starting from August 27, 2023, through August 25, 2024.

Data sources

Google Trends

The Google Trends platform offers unrestricted utilization of time-series data associated with the search volumes of certain phrases on the Google search engine [11]. To quantify the relative search volume (RSV), the total search query activity has been transformed to a percentage scale ranging from 0% to 100%. A value of 100% represents the highest point in search volume for a particular subject during a certain time period. In this situation, an RSV value of 50 would suggest that the search phrase had a popularity level that is 50% of the popularity seen in the most popular week. A score of 0 indicates a lack of searches to display for this topic throughout the week. Through a concurrent search of several phrases, we successfully compared the RSV for various terms. This paper specifically examined GLP-1 receptor agonists for weight loss searches both locally in Iraq and worldwide.

Search strategy

The word "Ozempic" was chosen because of its extensive coverage in social media and web/print publications pertaining to this subject matter. A comparative search was performed targeting the phrases "Wegovy," "Saxenda," and "Mounjaro" to identify a novel combination GLP-1 agonist, tirzepatide. All search criteria were restricted to include just inquiries conducted inside Iraq and to be opposed within the global dataset. The information used for statistical purposes was examined for an interval of one year. 

Statistical analysis

The statistical analysis was carried out using IBM SPSS Statistics for Windows, Version 23.0 (IBM Corp., Armonk, NY). GraphPad Prism version 8 for Windows (GraphPad Software, Inc., California, USA) was used to create the graphical representation of the data. The Kolmogorov-Smirnov test was employed to examine the normality of the distribution of RSV scores based on geographical regions. By employing independent T-tests to evaluate the differences between the RSV descriptive statistics for RSVs include the mean together with the mean of differences, 95% confidence interval, and correlation coefficient (r). A relation was concluded to be statistically significant when the p-value was less than 0.05. The graphical representation of the RSV scores exhibits a temporal pattern. Temporal structures were shown in Google Trends visualizations to illustrate the concentration of interest in each individual location for a certain topic.

## Results

**Figure 1:**
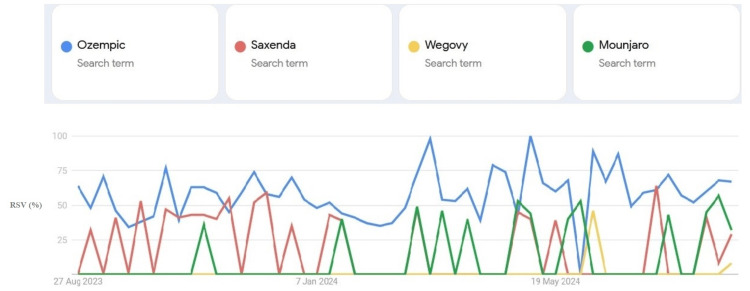
Ozempic, Saxenda, Wegovy, and Mounjaro 12-month relative search volume (RSV) report in Iraq

Google search interests for Ozempic, Saxenda, Wegovy, and Mounjaro from August 2023 to August 2024 in various areas of Iraq are displayed in Figure 1. Overall, Ozempic interest searches have spiked over time. Saxenda search was at its peak in the last months of 2023, whereas the search interest was almost flat for Wegovy and fluctuating for Mounjaro.

**Figure 2:**
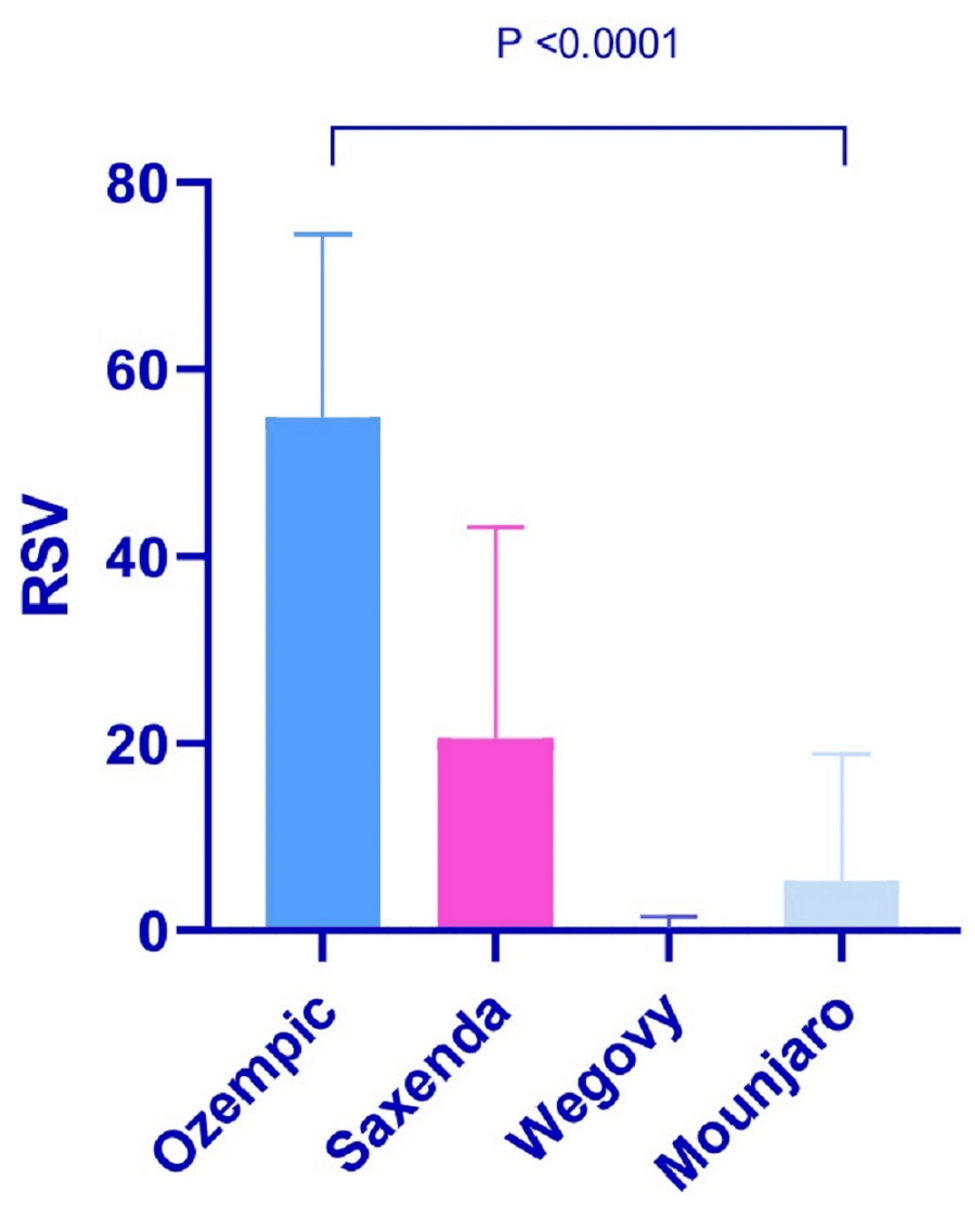
Ozempic, Saxenda, Wegovy, and Mounjaro relative search volume (RSV) mean difference in Iraq

Analysis findings indicated a statistically significant difference in the RSV across all compared pairs in Iraq search interest, as illustrated in Table 1 and Figure 2.

**Table 1:** Ozempic, Saxenda, Wegovy, and Mounjaro pair analysis for their search interest in Iraq

Pairs	Mean of differences	95% confidence interval	Correlation coefficient (r)	P-value
Ozempic versus Saxenda	-34.26	-43.47 to -25.06	-0.2450	<0.0001
Ozempic versus Wegovy	-54.57	-59.94 to -49.19	0.1392	<0.0001
Ozempic versus Mounjaro	-49.58	-56.58 to -42.59	-0.1336	<0.0001
Saxenda versus Mounjaro	-15.32	-22.53 to -8.110	0.0214	<0.0001
Saxenda versus Wegovy	-20.30	-26.51 to -14.09	0.09344	<0.0001
Wegovy versus Mounjaro	4.981	1.353 to 8.609	0.4150	0.0081

**Figure 3:**
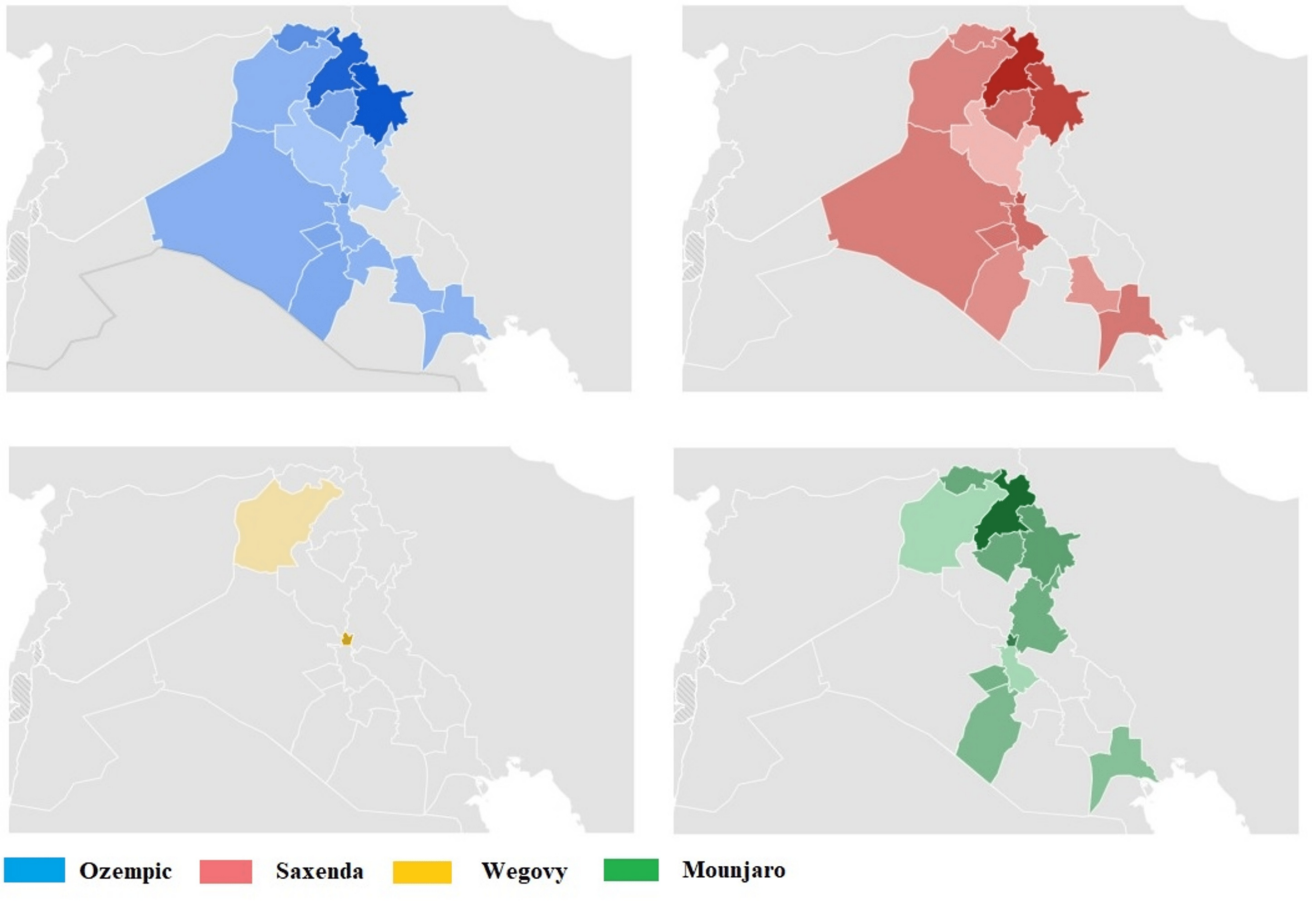
Ozempic, Saxenda, Wegovy, and Mounjaro relative search volume (RSV) in the Iraq subregion Darker shades indicate where the term has a higher probability of being searched.

**Figure 4:**
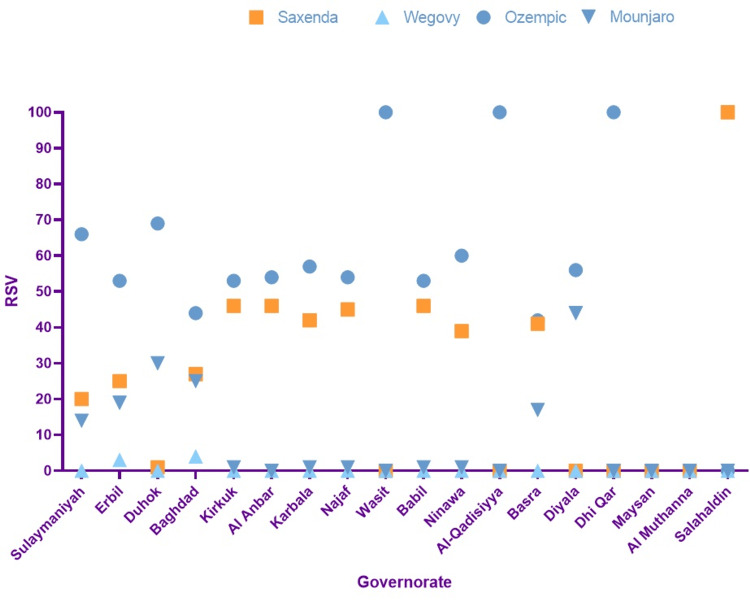
Ozempic, Saxenda, Wegovy, and Mounjaro relative search volume (RSV) numerical value in the Iraq subregion

The highest RSV for Ozempic was documented in Duhok with 69, followed by Sulaymaniyah with 66 and Erbil with 53. A comparable RSV profile is noted for Saxenda, while substantial interest in Wegovy is seen in Ninawa. The comprehensive details are shown in Figures 3-4.

The search phrases for the four products were not confined to their exact names; several inquiries have been documented, with some terms pertaining to pricing, side effects, and potency, while others were in Arabic, the native language of Iraq, as presented in Table 2.

**Table 2:** Documented search terms related to Ozempic, Saxenda, Wegovy, and Mounjaro in Iraq

Related search terms	RSV
How to use Mounjaro	5
Manjaro	3
Mounjaro injection	100
Mounjaro injection for weight loss	11
Mounjaro injection price	10
Mounjaro mechanism of action	<1
Mounjaro price	27
Mounjaro side effects	18
Mounjaro vs. Ozempic	11
Mounjaro weight loss	35
Mounjaro دواء	23
Ozempic weight loss	100
دواء Ozempic	20
Ozempic 0.25	37
Ozempic 0.25 mg	21
Ozempic 0.5	26
Ozempic 1 mg	73
Ozempic 18	18
Ozempic 1 mg	16
Ozempic dose	40
Ozempic dose for weight loss	18
Ozempic face	20
Ozempic for weight loss	52
Ozempic injection	56
Ozempic mechanism of action	13
Ozempic pen	90
Ozempic price	32
Ozempic side effects	55
Ozempic ابرة	45
Saxenda	49
Saxenda dose	21
Saxenda injection	32
Saxenda mechanism of action	4
Saxenda pen	45
Saxenda price	12
Saxenda side effects	27
Saxenda vs. Ozempic	19
Saxenda vs Ozempic	13
Saxenda علاج	100
Saxenda منحف	14
Semaglutide	25
Tirzepatide	8
Wegovy injection	62
Wegovy price	43
Wegovy vs. Ozempic	27
Wegovy سعر ابره	100
What is Ozempic	21
الاثار الجانبية Saxenda	39

**Figure 5:**
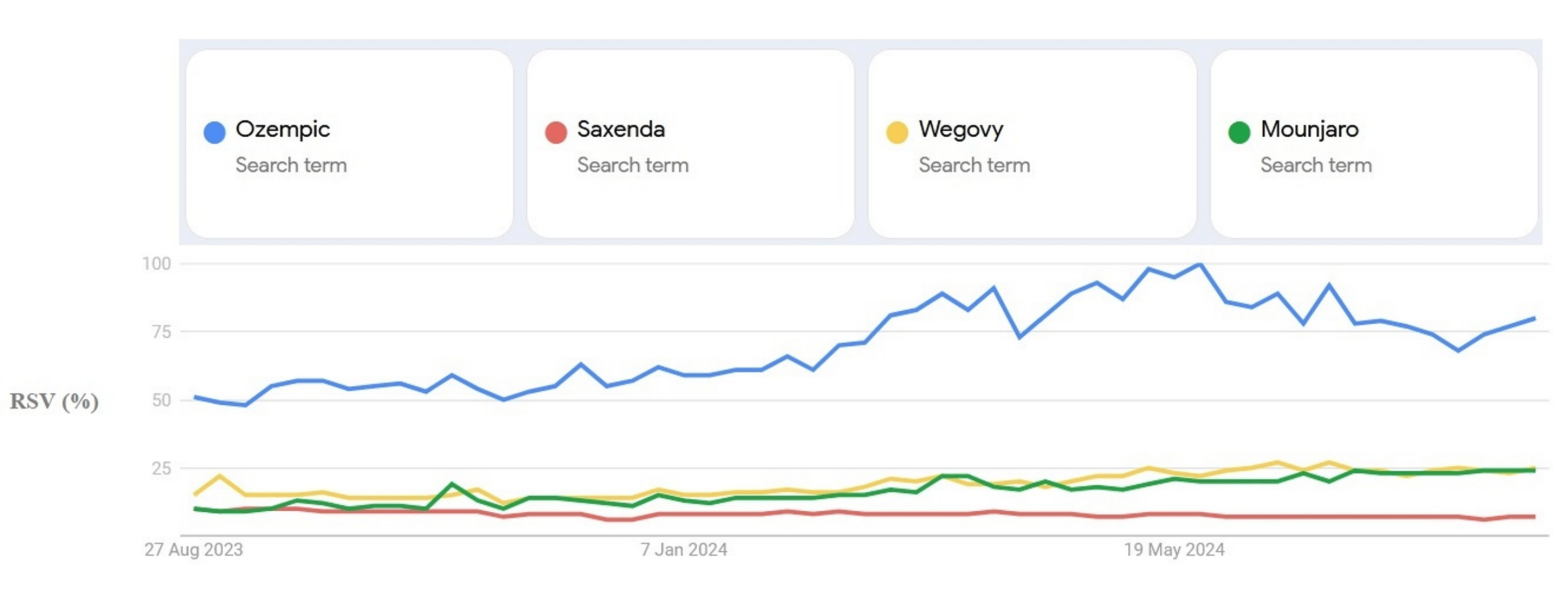
Ozempic, Saxenda, Wegovy, and Mounjaro global 12-month relative search volume (RSV) report

Figure 5 illustrates the global Google search interest for Ozempic, Saxenda, Wegovy, and Mounjaro from August 2023 to August 2024. Interest in Ozempic searches has increased over time. The search interest for Saxenda was the lowest among the four medications, whereas the interest for Wegovy and Mounjaro, although relatively low, demonstrated a gradual upward trend.

**Figure 6:**
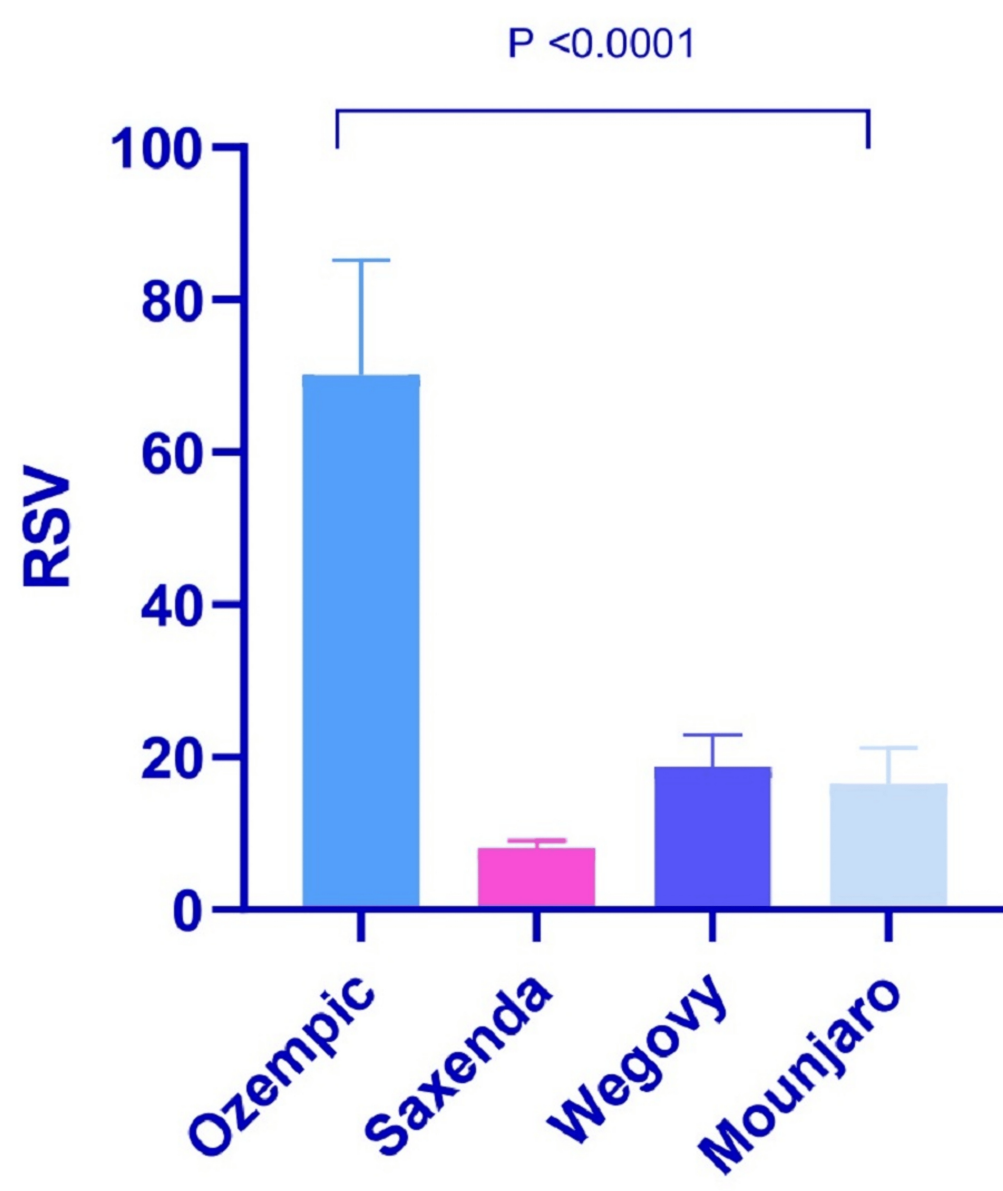
Ozempic, Saxenda, Wegovy, and Mounjaro relative search volume (RSV) mean difference in worldwide

Analysis findings indicated a statistically significant difference in the RSV across all compared pairs in global search interest, as illustrated in Table 3 and Figure 6.

**Table 3:** Ozempic, Saxenda, Wegovy, and Mounjaro pair analysis for their search interest worldwide

Pairs	Mean of differences	95% confidence interval	Correlation coefficient (r)	P-value
Ozempic vs. Saxenda	-62.19 ± 2.062	-66.28 to -58.10	0.8974	<0.0001
Ozempic vs. Wegovy	-51.45 ± 2.135	-55.69 to -47.22	0.8482	<0.0001
Ozempic vs. Mounjaro	-53.79 ± 2.160	-58.08 to -49.51	0.8564	<0.0001
Saxenda vs. Mounjaro	8.396 ± 0.6686	7.070 to 9.722	0.6026	<0.0001
Saxenda vs. Wegovy	10.74 ± 0.5820	- 9.582 to 11.89	0.7659	<0.0001
Wegovy vs. Mounjaro	-2.340 ± 0.8662	-4.057 to -0.6219	0.06555	0.0081

**Figure 7:**
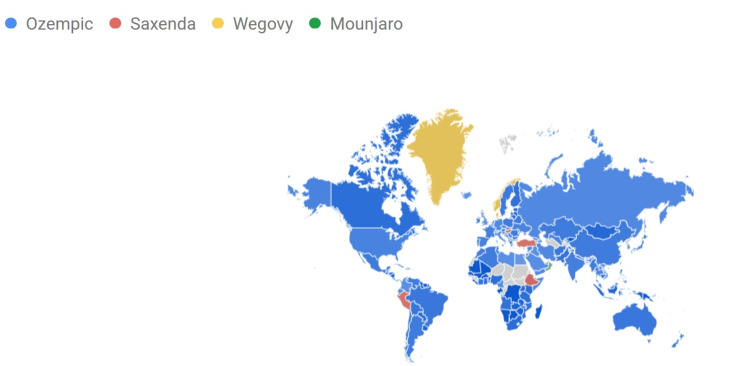
Ozempic, Saxenda, Wegovy, and Mounjaro RSV geographic distribution worldwide Darker shades indicate where the term has a higher probability of being searched.

The worldwide geographic distribution of interest is shown in Figure 7, which indicates a significant increase in searches for Ozempic over the last 12 months (August 2023-August 2024).

The worldwide search queries for the four drugs were not limited to their precise names; several questions were recorded, including keywords related to the scientific name, comparisons, costs, adverse reactions, and strength, as seen in Table 4.

**Table 4:** Ozempic, Saxenda, Wegovy, and Mounjaro related search terms documented worldwide

Related search terms	RSV
BMI	11
How to get ozempic	17
Liraglutide	11
Liraglutide saxenda	11
Mounjaro cost	21
Mounjaro coupon	36
Mounjaro diabetes	16
Mounjaro dose	18
Mounjaro for weight loss	48
Mounjaro injection	28
Mounjaro UK	18
Mounjaro vs. Ozempic	27
Mounjaro weight loss	100
Ozempic 1 mg	16
Ozempic buy	13
Ozempic cost	21
Ozempic diabetes	18
Ozempic dose	16
Ozempic face	36
Ozempic for weight loss	48
Ozempic injection	14
Ozempic Mounjaro	95
Ozempic online	16
Ozempic pen	18
Ozempic preã§o	15
Ozempic price	13
Ozempic Reddit	16
Ozempic side effects	76
Ozempic UK	13
Ozempic vs. Saxenda	17
Ozempic Wegovy	100
Ozempic weight loss	100
Preã§o Mounjaro	18
Preã§o Saxenda	21
Precio Saxenda	37
Reddit Mounjaro	16
Reddit Wegovy	12
Saxenda Australia	9
Saxenda cena	12
Saxenda dose	12
Saxenda for weight loss	15
Saxenda injection	18
Saxenda online	14
Saxenda Ozempic	100
Saxenda pen	19
Saxenda price	10
Saxenda side effects	20
Saxenda UK	14
Saxenda vs. Wegovy	13
Saxenda weight loss	43
Semaglutide Ozempic	24
Semaglutide Wegovy	21
Side effects Mounjaro	70
Side effects of Ozempic	15
Tirzepatide	20
Tirzepatide Mounjaro	21
Valor Saxenda	13
Victoza	19
Wegovy cost	22
Wegovy coupon	16
Wegovy dose	11
Wegovy for weight loss	21
Wegovy injection	11
Wegovy near me	11
Wegovy online	15
Wegovy price	10
Wegovy shortage	10
Wegovy side effects	37
Wegovy stock	13
Wegovy UK	15
Wegovy vs. Ozempic	22
Weight loss	98
Weight loss Wegovy	58
What is Mounjaro	30
What is Ozempic	45
What is Saxenda	13
What is Wegovy	22
Zepbound	18
Mounjaro drug	13
Mounjaro online	13

**Figure 8:**
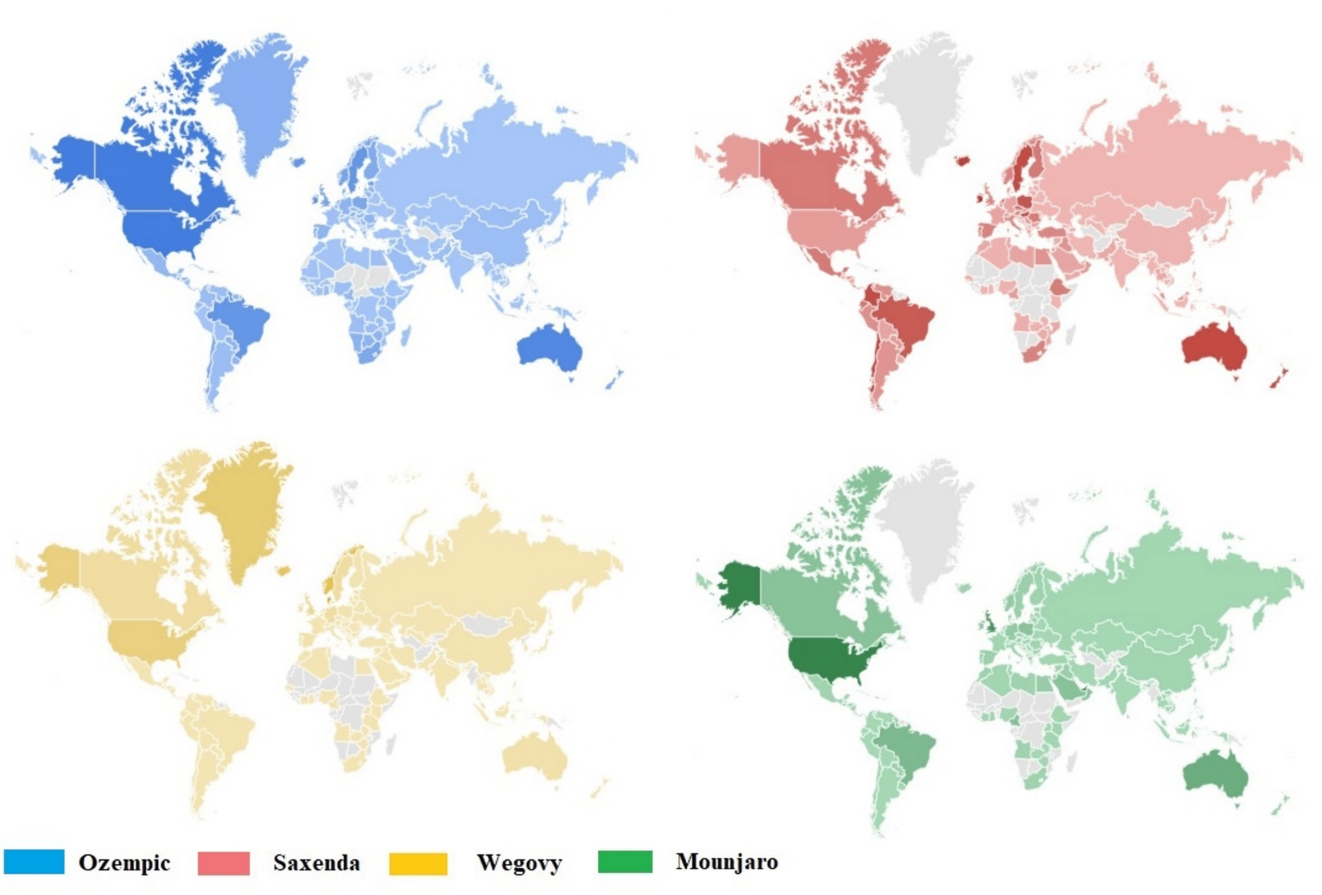
Ozempic, Saxenda, Wegovy, and Mounjaro RSV compared breakdown by region Darker shades indicate where the term has a higher probability of being searched.

The highest RSV for Ozempic was recorded in Canada, the United States of America, and Australia. A distinct RSV profile has been observed for Saxenda, with heightened search interest recorded in Latin America, Poland, Sweden, and Australia. By contrast, Wegovy received search interest primarily in Greenland and the United States, while Mounjaro's search interest was noted in Canada, the United States, and Australia. Figure 8 presents the entire information dissemination.

## Discussion

The research findings indicate a substantial rise in public interest in the search phrase “Ozempic, Saxenda, Wegovy, and Mounjaro related search terms” during the past 12 months in Iraq and the globe. GLP-1 agonists are recent additions to the healthcare sector, The US Food and Drug Administration first approved exenatide for type 2 diabetes mellitus in April 2005, subsequently bringing about the authorization of many more GLP-1 agonists [12]. In 2014, liraglutide became the initially approved GLP-1 agonist to get FDA clearance for weight loss in nondiabetic obese individuals [13]. In December 2017, Ozempic received approval for the management of type 2 diabetes mellitus. In June 2021, Wegovy received approval for the purpose of weight reduction in individuals with obesity or those who are overweight accompanied by at least one weight-related comorbidities. Mounjaro, a dual GLP-1 agonist and GIP (glucose-dependent insulinotropic polypeptide), received approval for use in the management of type 2 diabetes mellitus in May 2022 [14].

Wide-ranging social networking site recourse is frequently related to adverse self-perception regarding self-image and the onset of conditions such as depressive symptoms and eating habits. It has been argued that social media influencers and technologies like filters are responsible for an increase in certain complimentary cosmetic surgeries. A research study indicated that over 50% of participants undergoing nasal reconstruction were influenced by social networking sites "before and after" marketing campaigns [15]. Nonetheless, social media presents a significant opportunity for disseminating accessible health-informed advice aimed at mitigating obesity. Social media websites serve as an avenue of social assistance, which is essential for enhancing wellness across multiple illnesses. This could result in substantial weight loss due to social media-based programs for losing weight when compared to conventional strategies like leaflets or surveys [16]. Off-label prescriptions, particularly customized to the patient's specific demand, may be advantageous. Certain unapproved prescriptions have garnered an increasing amount of reliable information. Ozempic® at lesser dosages of 0.25, 0.5, and 1.0 mg is similar to Wegovy®, differing only in labeling and delivery method. Due to persistent supply chain challenges with Wegovy®, particularly affecting lower dosages, it may be beneficial to use Ozempic® off-label to mitigate these supply shortages. There is increasing evidence supporting the use of off-label antiobesity drugs, including metformin and topiramate [17].

Reviewers have raised reservations over off-label prescription usage of GLP-1 agonists. There is a significant worry over the dramatic increase in off-label prescriptions. Challenges arise from the few studies completed on the efficacy and safe administration of these drugs among adolescents. This may be ascribed to the latest authorization of many drugs for adults and the continuing studies [18]. A further problem is that the Food and Drug Administration prohibits direct-to-consumer marketing of off-label usage in patient awareness tools and commercials; nonetheless, off-label promotion by drug companies often triggers Medicaid false claim inspections [19]. There are concerns about the off-label use of these medications for those who do not meet the criteria of a BMI over 30 or a BMI over 27 with an accompanying weight-related disease. Numerous studies have shown effectiveness in individuals with diabetes and obesity; nevertheless, a concerning trend is the growing interest in Ozempic for aesthetic weight reduction, fueled by social media and celebrity endorsements. Moreover, off-label pharmaceuticals may interact with other drugs and exacerbate existing health disorders, and they may lack robust evidence to validate their safety and efficacy in both short and long-term scenarios across diverse patient groups [20].

Numerous limitations must be acknowledged when analyzing the findings of the current investigation. Initially, it is crucial to recognize that these results stem from an examination of consumer search habits and will probably impact by certain medication licenses and dissemination strategies. Second, these findings pertain only to search queries executed using the Google search engine. Despite Google's preeminence as a search engine, more research is required to ascertain how the search activity outlined above compares or contrasts with that of alternative search engines or networks. Social networking sites are now recognized as an important driver of spreading misconceptions about weight reduction. It is essential to acknowledge that the findings provided are derived from the total search volume assessed at the population level. Consequently, we cannot ascertain the extent to which alterations in Internet searching attitudes are attributable to variations in the number of individuals conducting a particular search, a spike in the frequency of searches by particular people, or an intersection of these elements.

## Conclusions

This study demonstrates a significant and growing public interest in GLP-1 agonists, namely, Ozempic, Saxenda, Wegovy, and Mounjaro. The search queries for the four drugs were not limited to their precise names; several queries have been recorded. In Iraq, the highest RSV for Ozempic was documented in Duhok, followed by Sulaymaniyah. A comparable RSV profile has been noted for Saxenda, while substantial interest in Wegovy is seen in Ninawa. Globally, the highest RSV for Ozempic was found in Canada, the United States of America, and Australia. Saxenda has a unique RSV profile, with increased search interest reported in Latin America, Poland, Sweden, and Australia. Wegovy, on the other hand, got the majority of searches in Greenland and the United States, while Mounjaro garnered searches in Canada, the United States, and Australia. As the use of GLP-1 agonists for weight loss becomes more common, more knowledge, understanding, and continuous scientific research will make it more convenient to obtain the best patient outcomes.
